# Oncocytic Papillary Cystadenoma: A Case of a Misdiagnosed Laryngeal Mass

**DOI:** 10.7759/cureus.52147

**Published:** 2024-01-12

**Authors:** Eftychia Kanioura, Dimitra Riga, Spyridon Potamianos, Georgia Kafiri

**Affiliations:** 1 Otorhinolaryngology Department, Hippokration General Hospital of Athens, Athens, GRC; 2 Pathology Department, Hippokration General Hospital of Athens, Athens, GRC

**Keywords:** cystadenoma, cyst, vocal cords, larynx, otorhinolaryngologic neoplasm, laryngeal disease, laryngeal neoplasms

## Abstract

Cystadenomas are benign neoplasms found in major and minor salivary glands. In cases where both oncocytic cells and papillary architecture, without a lymphoid component, exist, the lesion is called oncocytic papillary cystadenoma (OPC). OPCs are rarely encountered in the laryngeal region and that is why they are usually misdiagnosed as other types of laryngeal tumors. Hereby, we present a case of a misdiagnosed laryngeal OPC in an attempt to raise awareness of this rare entity, both for the surgeon performing the excision of the laryngeal mass and for the pathologists examining the specimen.

## Introduction

Cystadenomas are benign neoplasms found both in major and minor salivary glands [1]. They are rare entities, representing 4% of all salivary gland neoplasms and most commonly appear in women older than 40 years of age [1]. If both oncocytic cells and papillary architecture, without a lymphoid component, exist, the lesion is called oncocytic papillary cystadenoma (OPC) [1].

OPCs are most commonly encountered in areas where minor salivary glands are accumulated [1] while their presence in the laryngeal region is extremely rare [2,3]. Laryngeal OPCs were first described in 1897 by Koscher and Schaffer [3-5]. 

Hereby, we present the case of a laryngeal mass, originally misdiagnosed as a benign laryngeal cyst, which finally proved to be a laryngeal OPC. This case implies that laryngeal OPCs are usually misdiagnosed. Moreover, it indicates that surgeons should always bear in mind that laryngeal masses should be totally excised in order to avoid possible recurrence even if the gross appearance of a lesion is misleading as to its true nature.

## Case presentation

A 60-year-old woman presented to the Outpatient department of our hospital complaining of progressive voice hoarseness, as well as voice fatigue and dyspnoea, during the past three years. No symptoms of dysphagia, stridor, or weight loss were present. Her medical history was free of comorbidities. She was a heavy smoker of 50 pack-years. She also smoked cannabis for 10 years. No history of previous surgery in the head and neck area or intubation for other reasons was present.

During examination with a flexible fibreoptic endoscope (Olympus ENF-GP2 rhino-laryngo fiberscope; Olympus America Inc., Center Valley, PA, USA), a pedunculated, cystic, and fleshy mass, arising from the left true vocal cord was present, partially occluding the glottis through a valve mechanism. No enlargement of the mass with a Valsalva maneuver was noted. Both true vocal folds had normal mobility. No palpable neck mass was present. Because the mass was thought to be a benign cystic lesion, no further radiologic evaluation was conducted.

After a thorough patient consultation, the treatment was decided to be surgical excision of the mass. The patient was admitted to the hospital. Preoperative blood exams were between normal ranges. The rest of the preoperative evaluation was uneventful. During the microlaryngoscopy, a pedunculated mass initiating from the left vocal cord was noted. The mass was partially occluding the glottis without resulting in restriction of its movement. With the use of cold steel equipment, the mass was completely excised and the patient had an uneventful postoperative course. No drugs were administered to the patient perioperatively, apart from those required for the anesthesia. She was discharged from the hospital on the first postoperative day. After a four-month follow-up, no recurrence was noted and no voice hoarseness or voice fatigue was present.

**Figure 1:**
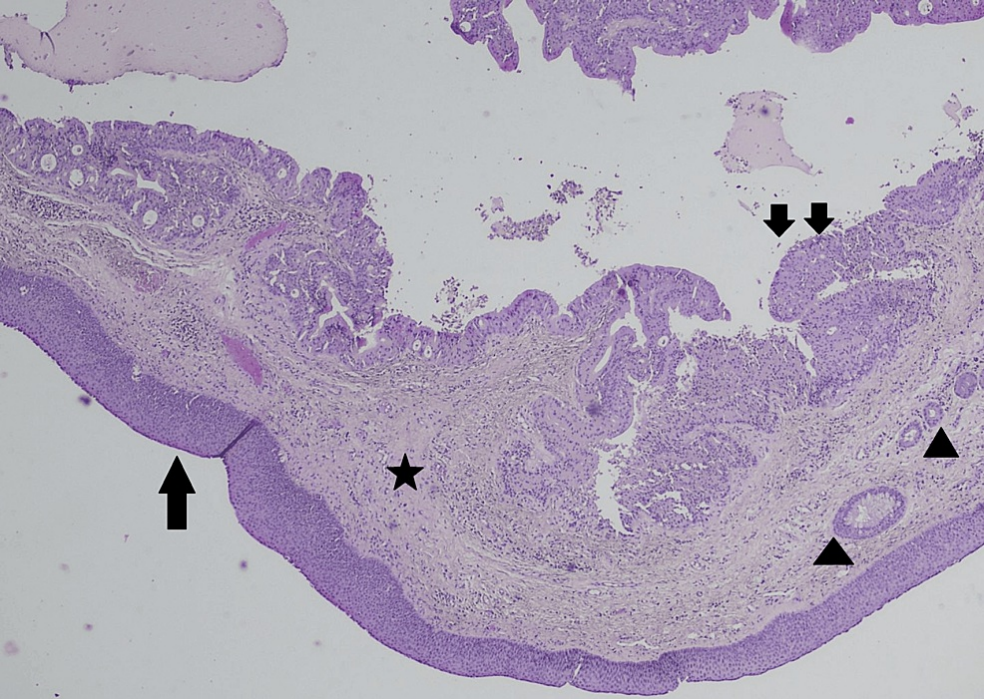
Hematoxylin-eosin, 10x Microscopic examination revealed a multicystic submucosal lesion, consisting of multiple cystic spaces. (single arrow: stratified squamous epithelium lining the true vocal cord, star: lamina propria, arrowhead: seromucinous glands in the lamina propria, double arrows: oncocytic papillary projections)

**Figure 2:**
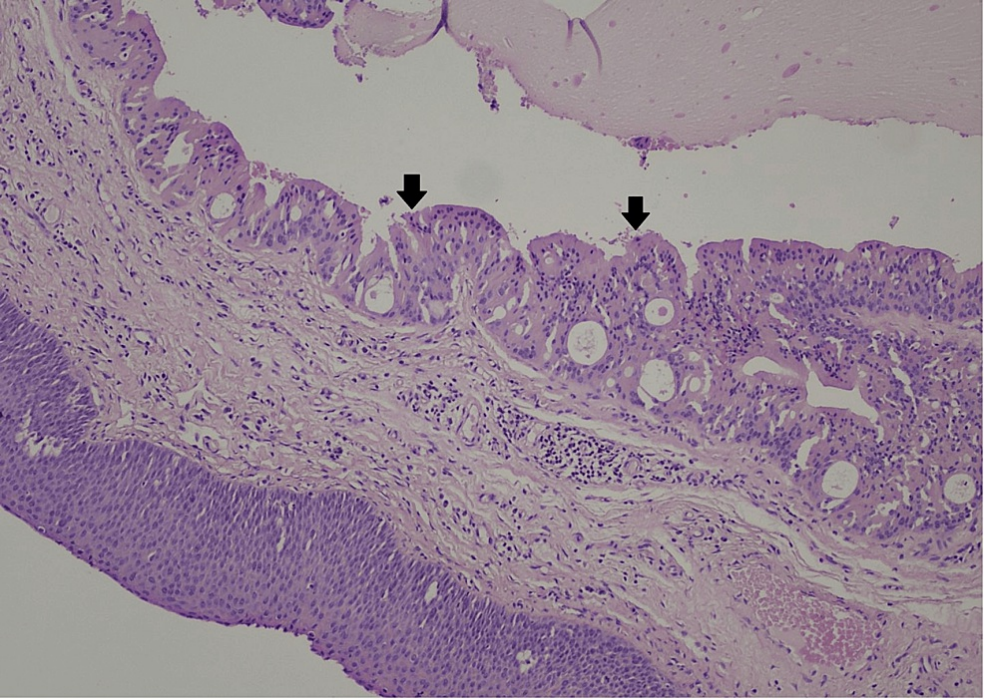
Hematoxylin-eosin, 20x The cystic spaces were lined by uniform oncocytic epithelial cells with intraluminal papillary projections. The oncocytic cells are epithelial cells with abundant eosinophilic, granular cytoplasm, and inconspicuous nucleoli, and without nuclear atypia. (The arrows indicate the papillary projections consisting of oncocytic epithelial cells)

The Pathology department of our hospital received five fibroelastic and fleshy tissue specimens, measuring from 0.2 cm to 1 cm. All tissue specimens were embedded into one paraffin block and the conventional hematoxylin-eosin staining method was used for the microscopic slides. Microscopic examination revealed a submucosal lesion, measuring 1 cm in greatest diameter, consisting of multiple cystic spaces (Figure 1) lined by epithelial columnar cells with abundant eosinophilic, granular cytoplasm, with inconspicuous nucleoli and without nuclear atypia, called oncocytic cells, with intraluminal papillary projections (Figure 2). No immunohistochemical stains were needed, as the morphologic characteristics of the cells were obvious at microscopic observation in a conventional hematoxylin-eosin stain.

**Figure 3:**
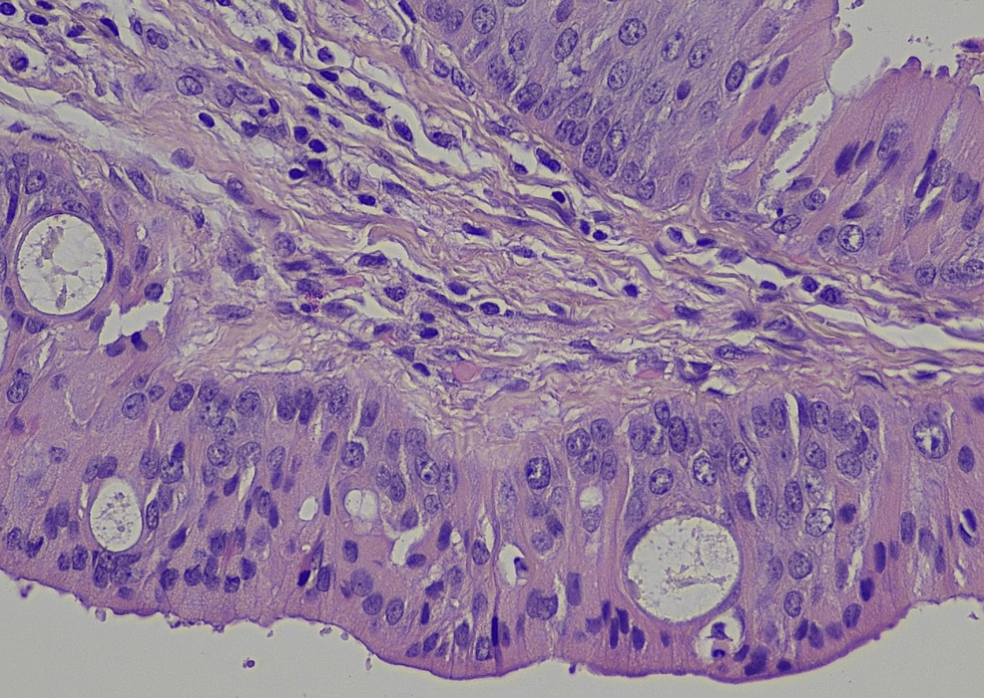
Hematoxylin-eosin, 40X On higher magnification, neither cellular atypia nor mitotic activity was observed.

**Figure 4:**
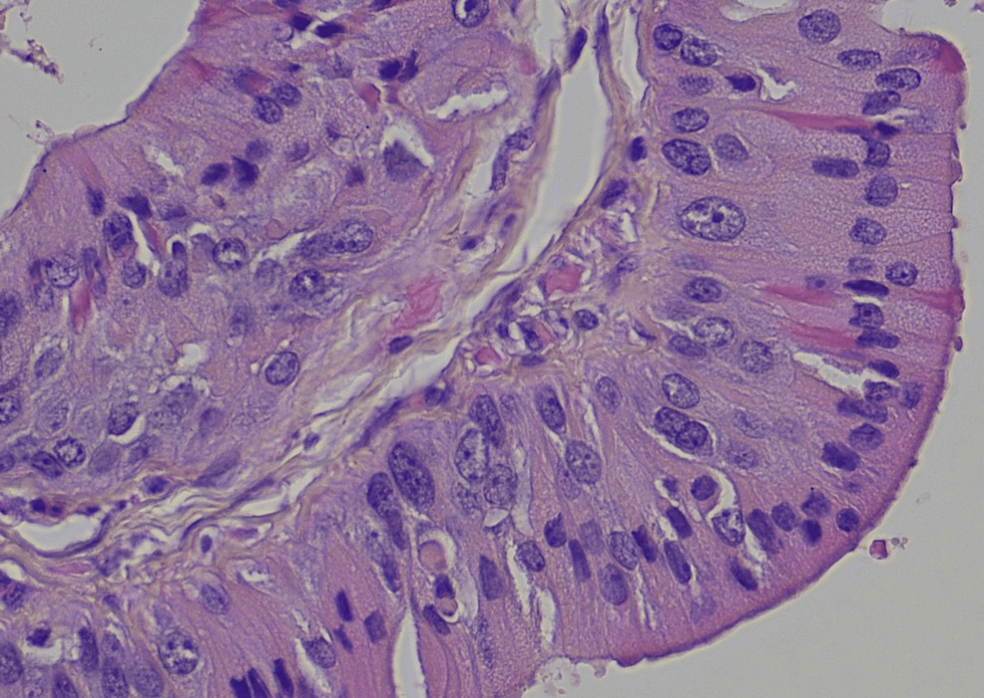
Hematoxylin-eosin, 60x The epithelial cells lining cystic spaces had uniform, normochromatic nuclei, with abundant granular oncocytic cytoplasm.

No atypia or mitotic activity was observed (Figures 3, 4).

The histopathologic diagnosis was oncocytic papillary cystadenoma of the true vocal cord.

## Discussion

OPCs are benign neoplasms rarely encountered in the head and neck region [6]. They have been reported to appear in the accessory tear glands, salivary glands, buccal mucosa, nasopharynx, oropharynx, and larynx [3,6]. Their presence in the larynx is extremely rare (less than 1% of all laryngeal tumors), accounting for approximately 150 cases in the literature [2,3,5,7,8]. As for the laryngeal subunits, they are most often encountered in the supraglottic region (74%), followed by the glottic (22%) and the subglottic region (4%) [2,3,5,8-10]. This is due to the higher accumulation of ductal epithelial cells in the supraglottic area [2,3,7,8].

The epithelium of the OPCs consists of oncocytes, which are large and irregularly shaped cells with granular eosinophilic cytoplasm rich in mitochondria [2,5,7-9]. The nuclei of the cells are hyperchromatic and the cytoplasm appears to be densely eosinophilic granular due to mitochondrial hyperplasia [2]. The origin of these cells is thought to be normal ductal or acinic cells that are being transformed due to inflammation or aging processes [2]. Smoking, as well as vocal fold comorbidities, are considered inflammatory risk factors that favor oncocytic metaplasia [2,3,7,10]. On the other hand, aging causes disturbance of mitochondrial enzyme organization, resulting in high metabolic activity and thus metaplasia of the epithelial cells [3]. It is quite interesting that, in our case, the patient was a frequent cannabis smoker. Despite the fact that smoking is correlated to cellular metaplasia, no literature report exists on the effect of cannabis on the oncocytic metaplasia of the cells.

As for the epidemiologic data, OPCs show a higher prevalence in older patients between the sixth and eighth decades of life, as well as a female predominance [2-5,7,8,10]. Their clinical presentation is most usually initiated with voice hoarseness [2,7,9]. Other symptoms include foreign body sensation, odynophagia, dysphagia, otalgia, dyspnoea, stridor, and airway obstruction [2,3,7]. If the mass expands through the thyrohyoid membrane, extralaryngeal manifestations, such as neck masses, have been described [2]. In a single case, OPC caused critical airway obstruction leading to death [5].

It is not uncommon that during clinical examination OPCs resemble other pathologies of the larynx, leading to their being misdiagnosed as other entities. Differential diagnosis should include laryngoceles, Reinke’s edema, mucus retention cysts, extranodal malignant lymphomas, saccular cysts, haemangiomas, minor salivary gland tumors, peripheral neural tumors, and squamous cell carcinoma of the larynx [2,3,9]. Laryngocele is the most often misdiagnosis [3]. In our case, the OPC was misdiagnosed as a laryngeal cyst.

Despite their benign nature, OPCs should be totally excised in order to avoid recurrence [3-5,7,8]. In cases where the masses are bilateral or diffuse, there is a higher chance of recurrence [10]. No malignant transformation of OPCs has been reported, although a case of laryngeal carcinoma arising in a multifocal OPC has been reported [2,7,11].

Apart from misleading clinical evidence that makes it difficult for the clinician to achieve the right diagnosis preoperatively, pathologists should differentiate OPCs from Warthin tumors, also known as papillary cystadenoma lymphomatosum [2]. Both lesions form a cystic neoplasm consisting of oncocytic cells with variable papillary projections, but OPCs lack the subepithelial lymphatic tissue [2,3]. Moreover, pathologists should always include neuroendocrine carcinoma of the larynx in the differential diagnosis of such lesions, despite them being rare and challenging entities [10].

## Conclusions

Due to their benign nature and very rare coexistence with malignant tumors, OPCs are thought to be easy-to-treat masses. Patients have an uneventful postoperative course and a low rate of recurrence when the masses are totally excised. Still, it is challenging to differentiate OPCs from other benign laryngeal masses not only in the preoperative evaluation of the patients but also during the pathological examination of the specimen retrieved during surgery. Total surgical excision of the laryngeal masses is therefore mandatory, even if they seem to be benign. On the other hand, pathologists should bear in mind that OPCs can be found in the laryngeal region. This way, differential diagnoses between other types of oncocytomas could be facilitated.
